# Machine-Learning Prediction of Oral Drug-Induced Liver Injury (DILI) via Multiple Features and Endpoints

**DOI:** 10.1155/2020/4795140

**Published:** 2020-05-19

**Authors:** Xiaobin Liu, Danhua Zheng, Yi Zhong, Zhaofan Xia, Heng Luo, Zuquan Weng

**Affiliations:** ^1^Department of Burns, Changhai Hospital, Second Military Medical University, Shanghai, China; ^2^The Centre for Big Data Research in Burns and Trauma, Fuzhou University, Fujian Province, China; ^3^College of Biological Science and Engineering, Fuzhou University, Fujian Province, China

## Abstract

Drug discovery is a costly process which usually takes more than 10 years and billions of dollars for one successful drug to enter the market. Despite all the safety tests, drugs may still cause adverse reactions and be restricted in use or even withdrawn from the market. Drug-induced liver injury (DILI) is one of the major adverse drug reactions, and computational models may be used to predict and reduce it. To assess the computational prediction performance of DILI, we curated DILI endpoints from three databases and prepared drug features including chemical descriptors, therapeutic classifications, gene expressions, and binding proteins. We trained machine-learning models to predict the various DILI endpoints using different drug features. Using the optimal feature sets, the top-performing models obtained areas under the receiver operating characteristic curve (AUC) around 0.8 for some DILI endpoints. We found that some features, including therapeutic classifications and proteins, have good prediction performance towards DILI. We also discovered that the severity of DILI endpoints as well as the selection of negative samples may significantly affect the prediction results. Overall, our study provided a comprehensive collection, curation, and prediction of DILI endpoints using various drug features, which may help the drug researchers to better understand and prevent DILI during the drug discovery process.

## 1. Introduction

The drug discovery process is both time-consuming and costly. It typically takes 10-17 years and costs $2.6 billion to develop a new drug [1, 2]. Even after a drug passes all the clinical trials and enters the market, it can still cause adverse drug reactions, which may result in restricted uses or even withdrawal [3, 4]. In the history of drug development, drug-induced liver injury (DILI) is one of the major factors to cause withdrawal of new drugs [5–7]. As an effort to reduce DILI, researchers have developed computational models to predict it [8, 9]. Machine learning is a method that utilizes computing systems to learn from the data and make predictions without the need of explicit programming [10]. Various machine-learning algorithms have been used to predict DILI, including *k*-nearest neighbor (KNN) [11, 12], Bayesian models [13, 14], linear discriminant analysis (LDA) [15], random forest (RF) [11, 16], support vector machine (SVM) [11], and artificial neural networks(ANN) [15]. Since predicting DILI may help to improve drug safety and reduce loss, this field is attracting interests from both the academia and the pharmaceutical industry.

However, predicting DILI is a challenging task since DILI involves different types of mechanisms such as direct hepatotoxicity, immune reactions, and mechanisms that are not completely understood [17, 18]. Besides, there are several limitations regarding the current approaches of DILI prediction. First, many studies focused on predicting either a single DILI endpoint or a superset of endpoints such as liver enzyme disorders, cytotoxic injury, cholestasis and jaundice, bile duct disorders [19], and liver steatosis [20]. Second, many studies focused on drug structural features [9, 12, 21, 22], while many additional types of data, such as binding assays [23], genomics [11], and postmarket surveillance data [19], are available. In this study, we collected a comprehensive dataset across different label sources (Micromedex DrugDex, Micromedex DrugPoints, and DailyMed), different feature types (chemical structure, protein binding, gene expression, and therapeutic classifications), and different DILI endpoints (such as liver failure, jaundice, biomarker increase, hepatomegaly, and hepatitis) for oral drugs. We investigated and evaluated model performance using different features to predict various DILI endpoints. We believe our results provide useful insights regarding DILI prediction and may potentially help to improve drug safety.

## 2. Methods

### 2.1. Feature Collection and Processing

**Figure 1:**
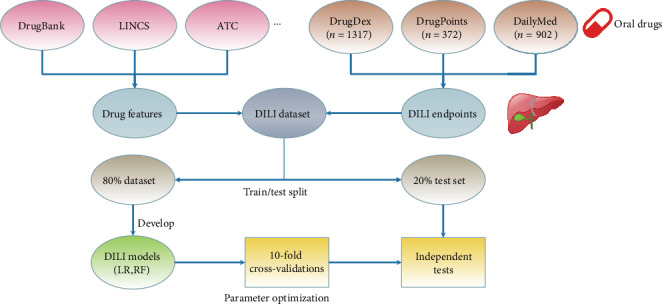
The workflow of this study. We collected drug features from various databases including DrugBank, LINCS, and WHO's ATC database and curated DILI labels from DrugDex, DrugPoints, and DailyMed for oral drugs. We split 20% of the dataset as an independent test set and used the remaining 80% for ten-fold cross-validations. We generated or collected the drug features and developed two types of models, logistic regression (LR) and random forest (RF), using different combinations of parameters and used the best parameters for independent tests.

The workflow of this study is shown in Figure 1. We collected multiple types of drug features from a variety of databases. The molecular weights and structures (SMILES format) of the drug molecules were collected from the PubChem database [24]. For structural features, we calculated five types of molecular descriptors including constitutional descriptors, electronic descriptors, geometrical descriptors, hybrid descriptors, and topological descriptors and three types of commonly used chemical fingerprints, including ECFP6 (1024 bits), PubChem fingerprints (881 bits), and standard fingerprints (1024 bits) using the rcdk package [25]. We collected the Anatomical Therapeutic Chemical (ATC) classification and Defined Daily Dose (DDD) codes from the World Health Organization (WHO). For protein binding features, the drug targets, enzymes, transporters, and carriers were collected from the DrugBank database [26]. For gene expression features, the drug-induced gene expression data for 978 landmark genes were collected from Wang et al. [27] based on the NIH Library of Integrated Network-Based Cellular Signatures (LINCS) database.

For feature processing, we categorized some continuous features into bins referring previous studies [28]. For example, the drug daily doses (DDD) were binned into DDD < 10 mg, 10 mg ≤ DDD < 100 mg, and DDD ≥ 100 mg. The solubility AlogP values were grouped into AlogP < 1, 1 ≤ AlogP < 3, and AlogP ≥ 3.

### 2.2. Endpoint Data Collection

The relationship between oral drugs and different types of DILI endpoints was extracted and curated from three databases, DrugDex, DrugPoints, and DailyMed, referring the extraction methods and criteria from previous studies [28]. For DrugDex, we extracted seven types of hepatic adverse drug reaction (hADR) endpoints including fatal hADRs, hADRs causing acute liver failure (liver failure), hADRs resulting in liver transplantation (liver transplantation), jaundice, biomarker increase, hepatomegaly, and hepatitis. The seven hADR endpoints were then categorized into severe hADRs (including fatal hADRs, liver failure, liver transplantation, and hADRs complying with Hy's law [29]) and less severe hADRs (including the rest hADRs). We ended up collecting 1,317 drugs from DrugDex for the above DILI endpoints (Supplementary Table 1). For DrugPoints, we collected endpoints including fatal hADRs, liver failure, jaundice, liver enzymes abnormal, bilirubin, hepatomegaly, and hepatitis for 372 drugs (Supplementary Table 2). The seven endpoints were also categorized into severe hADRs (including fatal hADRs and liver failure) and less severe hADRs (including the rest hADRs). For DailyMed, drugs were categorized into three groups: most concern, less concern, and no concern regarding DILI outcomes [30]. A drug is categorized as most concern for DILI when it was withdrawn from the market or given a warning, such as a black box warning or a precaution section of DILI; a drug is considered less concern for DILI if its label mentioned other DILI risks less severe than the previous criteria; and a drug with no concern for DILI does not have a DILI-related description in its label. We collected 902 drugs and 104, 235, and 563 of these drugs were categorized as most concern, less concern, and no concern for DILI, respectively (Supplementary Table 3).

For each endpoint, we defined two types of negative samples, NSap1 and NSap2. For a given hADR endpoint, NSap1 is defined as drugs that have no reported hepatotoxic reaction for the specific endpoint, while NSap2 is defined as drugs that have no reported hepatotoxic reaction across all endpoints. According to these definitions, NSap2 is a “cleaner” subset of NSap1.

### 2.3. Model Training and Assessment

For each dataset, we randomly held 20% as an independent test set and used the remaining 80% for training and validation. In this study, we trained two classifiers, logistic regression and random forest, using the scikit-learn package in Python. To minimize the data imbalance problem, the “class weight” parameter of each model was set to “balanced.” For each classifier, the best model parameters were selected by grid search based on areas under the receiver operating characteristic curve (AUC) during 10-fold cross-validations. Then, the model with the best parameters was evaluated on the independent test set.

Since we have two types of negative samples, NSap1 and NSap2, to find out whether the two types of negative samples had an impact on the model performance, we performed paired *t*-tests on the AUC scores of all features. We also ran paired *t*-tests specifically for the protein and ATC code features to find out whether they had any impact on the model performance.

## 3. Results and Discussion

### 3.1. Different Features and Model Performance

**Figure 2:**
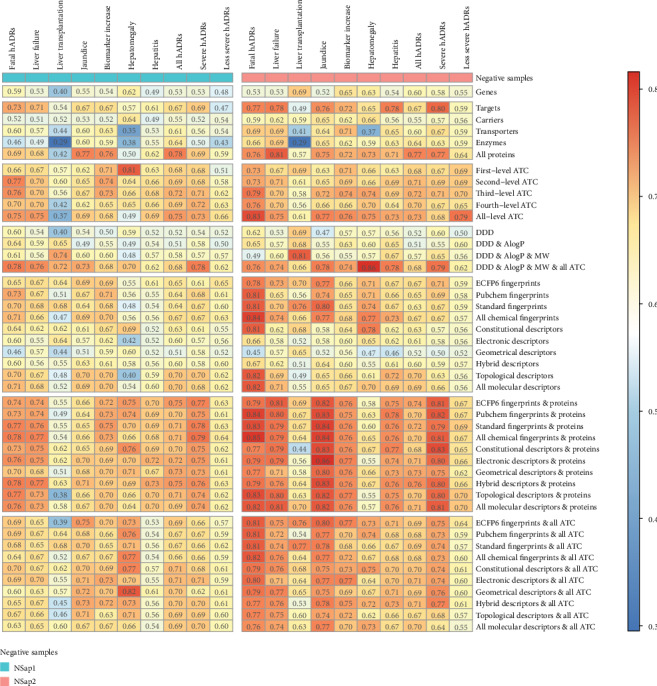
AUC values of different sets of features and DILI endpoints using random forest for drugs in the DrugDex database during 10-fold cross-validations. In the table, each row represents a set of drug features, each column represents a DILI endpoint and the negative sample set (NSap1 vs. NSap2), and each cell represents an AUC value (colored by its value). For DrugDex, there are seven DILI endpoints (fatal hADRs, liver failure, liver transplantation, jaundice, biomarker increase, hepatomegaly, and hepatitis). They were categorized as “severe hADRs” and “less severe hADRs.”. “All hADRs” include all DILI endpoints.

We trained two types of classifiers, logistic regression and random forest, to predict different DILI endpoints using different types of features for drugs in the DrugDex, DrugPoints, and DailyMed databases. 10-fold cross-validations and independent tests were conducted to estimate model performance on the three databases. The AUC values of 10-fold cross-validations on the datasets using best parameters were visualized by heat map in Figure 2 and Supplementary Figs. 1–5. The results of the independent tests are in Supplementary Tables 4-6. Since some endpoints have very few or zero positive samples during the independent test and produced abnormally high or zero AUC values, we focused our analysis based on the results of 10-fold cross-validations and provided the independent test results as additional references in Supplementary Tables 4-6.

Like the previous study [31], we used different types of chemical fingerprints to predict DILI. While the logistic regression models showed random performance (AUC = 0.5) on most endpoints using chemical fingerprints as features, the models got slightly better performance for the “All hADR” endpoint on either the NSap1 or NSap2 dataset with AUC values mostly larger than 0.6 (Supplementary Figure 1). For random forest models, the performance is generally better than logistic regression models using chemical fingerprints, especially for endpoints like fatal hADRs and severe hADRs, which have AUC values close to 0.8 (Figure 2). Similar results were also found for endpoints in DrugPoints and DailyMed. Since random forest is an ensemble model with a more complex structure, it is expected that it exceeded the performance of logistic regression. The models showed similar performance patterns using molecular descriptors as features, with a few exceptions.

ATC codes are hierarchical therapeutic classifications of drugs. A previous study has identified associations between drug indications and side effects [32]; thus, we assumed that the therapeutic classifications might also be helpful in predicting DILI. From the results, we can see that ATCs have better performance for predicting most DILI endpoints compared to chemical fingerprints. The logistic regression and random forest models using the second level to fourth level of ATC codes were able to obtain AUC values around or larger than 0.7 in most DILI endpoints. However, the first level of ATC codes had worse performance due to a lack of therapeutic classification details. We also combined ATC codes with other features, including the chemical fingerprints and molecular descriptors. We found that the combination generally improved the model performance than using a single type of features, indicating the usefulness of combining various types of features (Figure 2 and Supplementary Figs. 1-5).

According to the DILIN prospective study [33], drugs in specific categories may have a higher association with DILI, as the authors indicated 45% of the 899 investigated DILI cases were caused by antimicrobials. To find out if similar patterns can be observed in our data, we took drugs collected from DrugDex as an example and calculated the odds ratio (OR) and Fisher's exact test *p* values between their top-level ATC codes and different DILI endpoints. The results are shown in Supplementary Table 7. We observed that for anti-infective drugs for systemic use, their odds ratios against all DILI endpoints are above 2.5 with *p* values < 0.01, indicating a significant positive association. We also analyzed the feature importance for prediction (Supplementary Table 8) and found this category was relatively important to predict various DILI endpoints, which is consistent with the previous study. Additionally, we observed that antineoplastic and immunomodulating agents and drugs for the musculoskeletal system may also have a higher association with DILI compared to drugs in other categories. We believe such data and analysis can provide valuable information to understand and prevent DILI.

The gene expression features used in this study [27] represent gene expression changes of the LINC L1000 978 landmark genes aggregated from a variety of cell lines before and after treatment by drugs. The results showed that their AUC values ranged mostly between 0.5 and 0.6 in all three databases. This indicates that the processed dataset of LINCS gene expression profiles may not be good enough to predict DILI, possibly because the immortal cell lines in which drugs were tested may not necessarily represent the specific cell types of hepatocytes or liver tissues. Thus, the expression profiles aggregated from these experiments may not be predictive towards DILI endpoints.

To explore the importance of protein features in predicting DILI, we trained models to predict various DILI endpoints using drug-binding proteins including targets, carriers, transporters, and enzymes. We found that using a single type of protein features alone, the models obtained various results with the highest AUC value around 0.8. Meanwhile, combining all types of protein features could improve model performance even more. Additionally, we found combining the protein features with the chemical fingerprints or molecular descriptors could significantly improve the performance of just using chemical fingerprints or molecular descriptors in most cases of DrugDex and DrugPoints and some cases of DailyMed (Table 1). This indicates the protein-binding profiles of drugs are potentially important indicators for DILI. Liu et al. [34] investigated the prediction of adverse drug reactions using chemical features, protein features, and phenotypic properties of drugs. They also found that the combination of both protein features and chemical features improved the prediction performance compared to using only one of them. As one family of adverse drug reactions, DILI has idiosyncratic and complicated mechanisms [18]. Since protein features provide important target-binding information in addition to chemical features, we believe the combination of such multidimensional data can improve the model prediction performance.

### 3.2. Network and Pathway Analysis of Protein Features

**Figure 3:**
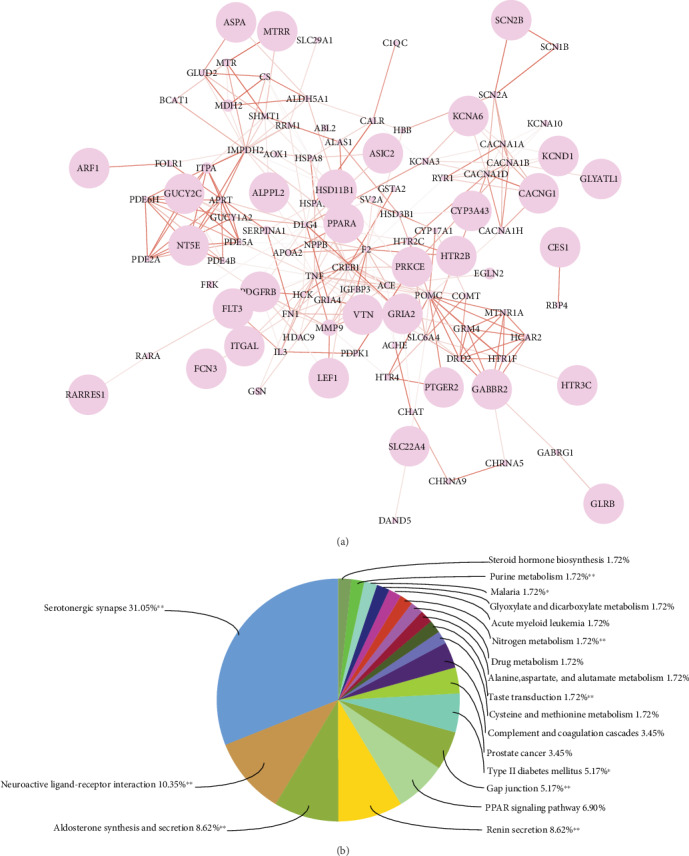
For fatal hADRs as the endpoint, (a) the network of proteins according to the feature importance and (b) KEGG pathway analysis of important protein features. In (a), each protein is represented by its gene symbol. The node size represents feature importance of protein to DILI models. The line thickness presents the combined score made by the STRING database. In (b), the important protein features were selected and analyzed by Cytoscape ClueGO using KEGG pathways. The stars indicate the significance levels for the enrichment tests.

In this section, we did network and pathway analyses of the protein features using the DrugDex database as an example. To find out which proteins and pathways are important to DILI prediction, we calculated the Gini importance values for the protein features using ExtraTrees [35]. For each endpoint, we selected proteins with feature importance equal or larger than 0.001 and queried the STRING database [36] to find the protein-protein associations among them. The protein-protein association networks are visualized in Figure 3(a) and Supplementary Figure 6 indicating protein-protein binding, coexistence in the same functional pathway/process, or other indirect interactions. From Figure 3(a), we found that some highlighted genes, such as PPARA, HTR2B, and SLC22A4, were reported in the literature to be associated with DILI or liver diseases [37–39]. We believe this feature analysis may provide helpful insights to identify potential DILI-related genes and generate new hypotheses to be further tested in the wet lab.

We also used the ClueGO plugin in Cytoscape [40, 41] to explore which pathways are enriched among the proteins passing our feature importance criteria (Figure 3(b) and Supplementary Figure 6). We found that the serotonergic synapse pathway was significantly enriched for fatal hADRs and the dopaminergic synapse pathway was significantly enriched for a few other DILI endpoints. Studies showed that serotonin and dopamine may have an association with neuropsychiatric symptoms and neurobiology of liver failure [42, 43]. From our analysis, we believe the feature importance analysis and pathway enrichment analysis may help to generate new hypotheses and useful insights for the DILI mechanisms and thus aid in the understanding and prevention of DILI.

### 3.3. Different Endpoints and Model Performance

We compared the AUC values of all the features between the endpoints of severe hADRs and less severe hADRs and found the models mostly performed better on severe hADRs (Table 2). We also observed better performance on endpoints of fatal hADRs and liver failure compared to other endpoints (Figure 2 and Supplementary Figs. 1-5). It is suggested that these severe DILI endpoints are more predictable than less severe endpoints. Interestingly, as an exception, the jaundice endpoint which belongs to less severe hADRs was found to be predicted well using protein features. This finding is consistent with a previous study which showed the importance of transporters in the cholestasis model [44].

### 3.4. Negative Sample Selection and Model Performance

To elucidate the differences of selecting negative samples in DILI model performance, we prepared two types of negative drugs in three databases, NSap1 and NSap2. In general, the models performed better using NSap2 as negative samples compared to NSap1 (Figure 2 and Supplementary Figs 1-5). Paired *t*-test results of the AUC values in each endpoint between NSap1 and NSap2 are shown in Table 3. We found that for most endpoints in DrugDex, using NSap2 as negative samples had better results than using NSap1. Thus, the selection of negative samples could make a significant difference in predicting DILI endpoints.

Defining an accurate negative set is important to study DILI; however, different sources may lead to different negative sets. Zhu and Li [45] identified a set of 957 drugs without hepatotoxicity report from eHealthMe websites as the negative set, which was also used in the work of Bajzelj and Drgan [46]. DILIrank [47] contains a negative set of 312 no-DILI-concern drugs whose labels did not contain any DILI indication, and this set was later used in the study of Shin et al. [48]. He et al. [49] collected a negative set of 709 drugs without hepatotoxicity records from various literature sources. Note that all the above approaches are similar to our approach, which is to define drugs without reported hepatotoxic reaction as the negative set. However, since different research groups utilized different sources to determine their negative sets, it can be challenging to find a consistent gold standard. Taking DILIrank [47] as an example, while 38% of its no-DILI-concern drugs also exist in our negative set collected from DrugDex, a lower proportion (31%) was found in the negative set from Zhu and Li [45].

## 4. Conclusions

In this study, we collected different types of drug features, including chemical fingerprints, molecular descriptors, binding proteins, gene expression, and therapeutic classifications, and collected the DILI endpoints from three databases, DrugDex, DrugPoints, and DailyMed. We trained machine-learning models to predict the DILI endpoints using the various features. The models were assessed via 10-fold cross-validations, and the results were analyzed by different types of features and endpoints. We found that

1. the features of ATC codes or binding proteins may have significant implications for prediction performance. Analyzing the important protein features using networks and pathways may elicit potential insights regarding DILI mechanisms
2. severe liver injury, such as fetal hADRs, severe hADRs, and liver failure, had better prediction performance compared to nonsevere endpoints
3. the selection of negative samples had an impact on DILI prediction. Clean negative samples of drugs without any DILI information in their labels may produce better performance for DILI predictions

We also provided all the curated DILI labels from three databases. We believe our study provides valuable information and comprehensive evaluations for computational DILI prediction and may help researchers to better understand DILI and improve drug safety.

## Figures and Tables

**Table 1:** Paired *t*-test results of AUC values during 10-fold cross-validations with or without using protein-binding features.

		Logistic regression		Random forest	
Database	Features	*t*	*p*	*t*	*p*
DrugDex	ECFP6 fingerprints	-3.51	1.96*E*-03∗∗	-2.48	1.80*E*-02∗
	PubChem fingerprints	-3.09	5.38*E*-03∗∗	-2.56	1.48*E*-02∗
	Standard fingerprints	-3.32	2.86*E*-03∗∗	-2.26	2.94*E*-02∗
	Constitutional descriptors	-2.12	4.35*E*-02∗	-2.96	5.41*E*-03∗∗
	Electronic descriptors	-4.44	1.14*E*-04∗∗	-6.10	7.04*E*-07∗∗
	Geometrical descriptors	-5.75	4.22*E*-06∗∗	-8.30	6.47*E*-10∗∗
	Hybrid descriptors	-3.50	1.90*E*-03∗∗	-8.79	5.96*E*-10∗∗
	Topological descriptors	-2.35	2.43*E*-02∗	-1.93	6.11*E*-02
	All fingerprints	-2.34	2.68*E*-02∗	-1.94	5.95*E*-02
	All descriptors	-2.63	1.29*E*-02∗	-2.48	1.78*E*-02∗
	All combined	-10.25	2.76*E*-21∗∗	-10.56	3.79*E*-23∗∗

DrugPoints	ECFP6 fingerprints	-2.06	5.60*E*-02	-2.99	8.91*E*-03∗∗
	PubChem fingerprints	-3.26	9.78*E*-03∗∗	0.10	9.19*E*-01
	Standard fingerprints	-2.66	2.10*E*-02∗	-2.49	2.51*E*-02∗
	Constitutional descriptors	-3.20	4.97*E*-03∗∗	-2.18	4.28*E*-02∗
	Electronic descriptors	-3.31	5.00*E*-03∗∗	-3.51	2.98*E*-03∗∗
	Geometrical descriptors	-5.42	4.06*E*-05∗∗	-5.21	6.70*E*-05∗∗
	Hybrid descriptors	-4.80	9.79*E*-04∗∗	-2.31	3.55*E*-02∗
	Topological descriptors	-4.04	8.19*E*-04∗∗	-3.04	7.08*E*-03∗∗
	All fingerprints	-2.41	2.75*E*-02∗	-2.03	5.80*E*-02∗
	All descriptors	-4.61	3.56*E*-04∗∗	-2.35	3.08*E*-02∗
	All combined	-10.13	2.42*E*-19∗∗	-7.30	1.04*E*-11∗∗

DailyMed	ECFP6 fingerprints	-0.79	4.50*E*-01	-0.31	7.62*E*-01
	PubChem fingerprints	-2.24	7.56*E*-02	-0.35	7.37*E*-01
	Standard fingerprints	0.00	1.00*E*+00	-0.85	4.19*E*-01
	Constitutional descriptors	-0.94	3.80*E*-01	-1.56	1.53*E*-01
	Electronic descriptors	-1.25	2.58*E*-01	-1.65	1.30*E*-01
	Geometrical descriptors	-2.10	8.66*E*-02	-4.80	7.95*E*-04∗∗
	Hybrid descriptors	-2.81	3.74*E*-02∗	-1.49	1.79*E*-01
	Topological descriptors	-0.27	7.97*E*-01	-0.26	8.00*E*-01
	All fingerprints	0.10	9.26*E*-01	-0.23	8.24*E*-01
	All descriptors	-0.90	3.97*E*-01	-0.56	5.87*E*-01
	All combined	-3.16	2.06*E*-03∗∗	-2.88	4.74*E*-03∗∗

For each *t*-test, the AUC score vectors of model performance on all endpoints were paired up and compared. ^∗^*p* < 0.05; ^∗∗^*p* < 0.01.

**Table 2:** Paired *t*-test results of AUC values during 10-fold cross-validations between severe hADRs and less severe hADRs using NSap2 as negative examples.

	Logistic regression		Random forest	
Database	*t*	*p*	*t*	*p*
DrugDex	2.51	1.77*E*-02∗	3.72	8.13*E*-04∗∗
DrugPoints	3.36	1.92*E*-03∗∗	1.73	9.18*E*-02
DailyMed	-0.07	9.45*E*-01	5.16	2.41*E*-05∗∗

For each endpoint, the AUC score vectors of model performance on all features were paired up and compared. ^∗^*p* < 0.05; ^∗∗^*p* < 0.01.

**Table 3:** Paired *t*-test results of AUC values during 10-fold cross-validations between NSap1 and NSap2 as negative examples.

		Logistic regression		Random forest	
Database	Features	*t*	*p*	*t*	*p*
DrugDex	Fatal hADRs	-3.80	7.69*E*-04∗∗	-2.83	7.53*E*-03∗∗
	Liver failure	-3.33	2.46*E*-03∗∗	-1.51	1.40*E*-01
	Liver transplantation	-2.33	2.63*E*-02∗	-2.50	1.69*E*-02∗
	Jaundice	-3.10	4.04*E*-03∗∗	-3.69	1.01*E*-03∗∗
	Biomarker increase	-2.76	9.05*E*-03∗∗	-0.59	5.60*E*-01
	Hepatomegaly	-0.35	7.28*E*-01	-0.72	4.77*E*-01
	Hepatitis	-3.15	3.52*E*-03∗∗	-3.00	4.70*E*-03∗∗
	All hADRs	-0.12	9.02*E*-01	-0.03	9.78*E*-01
	Severe hADRs	-3.65	1.06*E*-03∗∗	-0.68	5.00*E*-01
	Less severe hADRs	-2.74	9.73*E*-03∗∗	-0.58	5.65*E*-01

DrugPoints	Liver failure	-0.82	4.20*E*-01	0.42	6.75*E*-01
	Jaundice	-0.11	9.15*E*-01	1.18	2.47*E*-01
	All hADRs	-0.81	4.21*E*-01	0.04	9.67*E*-01
	Severe hADRs	-1.37	1.78*E*-01	-0.03	9.74*E*-01
	Less severe hADRs	0.85	4.01*E*-01	-0.41	6.81*E*-01

DailyMed	All hADRs	0.00	1.00*E*+00	0.00	1.00*E*+00
	Severe hADRs	5.22	6.75*E*-06∗∗	-0.60	5.50*E*-01
	Less severe hADRs	1.41	1.72*E*-01	10.04	1.57*E*-10∗∗

For each endpoint, the AUC score vectors of model performance on all features were paired up and compared. ^∗^*p* < 0.05; ^∗∗^*p* < 0.01.

## Data Availability

The data used to support the findings of this study are available from the article and supplementary information file.

## Supplementary Materials

Supplementary Materials Supplementary Figure 1: AUC values of different sets of features and DILI endpoints using logistic regression for drugs in the DrugDex database during 10-fold cross-validations. Supplementary Figure 2: AUC values of different sets of features and DILI endpoints using logistic regression for drugs in the DrugPoints database during 10-fold cross-validations. Supplementary Figure 3: AUC values of different sets of features and DILI endpoints using random forest for drugs in the DrugPoints database during 10-fold cross-validations. Supplementary Figure 4: AUC values of different sets of features and DILI endpoints using logistic regression for drugs in the DailyMed database during 10-fold cross-validations. Supplementary Figure 5: AUC values of different sets of features and DILI endpoints using random forest for drugs in the DailyMed database during 10-fold cross-validations. Supplementary Figure 6: for the other DILI endpoints in DrugDex, the network of proteins according to the feature importance (a), and KEGG pathway analysis of important protein features (b). Supplementary Table 1: DILI endpoints curated from DrugDex. Supplementary Table 2: DILI endpoints curated from DrugPoints. Supplementary Table 3: DILI endpoints curated from DailyMed. Supplementary Table 4: AUC values of different sets of features and DILI endpoints for drugs in the DrugDex database during an independent test. Supplementary Table 5: AUC values of different sets of features and DILI endpoints for drugs in the DrugPoints database during an independent test. Supplementary Table 6: AUC values of different sets of features and DILI endpoints for drugs in the DailyMed database during an independent test. Supplementary Table 7: association between DrugDex DILI endpoints and top-level ATC codes. Supplementary Table 8: feature importance of using top-level ATC codes to predict DrugDex DILI endpoints.
